# Use of Plant and Herb Derived Medicine for Therapeutic Usage in Cardiology

**DOI:** 10.3390/medicines5020038

**Published:** 2018-04-22

**Authors:** Ye Eun Koo, Jiwon Song, Soochan Bae

**Affiliations:** Beth Israel Deaconess Medical Center, Cardiovascular Institute, 3 Blackfan Circle, CLS 910, Boston, MA 02215, USA; yeeun.koo@tufts.edu (Y.E.K.); jane.song@richmond.edu (J.S.)

**Keywords:** pharmacognosy, therapeutic, cardiology, antioxidant, anti-inflammatory

## Abstract

Cardiovascular diseases (CVDs) have become prominent in mortality and morbidity rates. Prevalent cardiovascular conditions, such as hypertension, atherosclerosis and oxidative stress, are increasing at an alarming rate. Conventional drugs have been associated with adverse effects, suggesting a need for an alternative measure to ameliorate CVD. A number of plant- and herb-derived preventative food and therapeutic drugs for cardiovascular conditions are progressively used for their various benefits. Naturally derived food and drugs have fewer side effects because they come from natural elements; preventative food, such as grape seed, inhibits changes of histopathology and biomarkers in vital organs whereas therapeutic drugs, for instance Xanthone, improve heart functions by suppressing oxidative stress of myocyte. This review closely examines the various plant- and herb-derived drugs that have assumed an essential role in treating inflammation and oxidative stress for prevalent cardiovascular conditions. Furthermore, the use of plant-derived medicine with other synthetic particles, such as nanoparticles, for targeted therapy is investigated for its effective clinical use in the future.

## 1. Introduction

Cardiovascular diseases (CVD) are often life threatening and pervasive in regards to health [1]. Some of the most prevalent types of CVD include hypertension, atherosclerosis, and myocardial ischemic reperfusion injury (IRI) [2,3,4]. To mitigate the detrimental effects of CVD, researchers have further investigated bioactivities in foods and plants [5]. Using plants for improving health has shown beneficial outcomes, as plants have long been a source of exogenous (i.e., dietary) antioxidants [6]. Approximately two-thirds of the world’s plant species are widely used in medicines, and almost all of these exhibit excellent antioxidant potential [7]. The antioxidant potential of plants has received a great amount of attention for increased oxidative stress has been identified as a major causative factor of CVD. Approximately 80% of the world population uses herbal medicines due to their low toxicity and better acceptability by the human body [8,9]. 

## 2. History of Plant- and Herb-Derived Medicine and Their Usage in Cardiology 

Adapting plants and herbs for medicinal application has a long history [10,11]. Humans began using plants for medicinal purposes as early as middle Paleolithic age, approximately 60,000 years ago, and now the World Health Organization estimates that over 80% of the people in developing nations rely on traditional remedies such as herbs, for food and healing sickness [11,12]. The use of medicinal plants has been developed over a long period of time and now plays a critical role in favorable health outcomes. Today, a number of pharmaceuticals currently approved by the Food and Drug Administration (FDA) have originated from plants; natural products (and their derivatives and analogs) represent over 50% of all drugs in clinical use [11,13,14]. 

Furthermore, plant-derived medicines have a long history of use for the prevention and treatment of CVDs [11]. Various industries are now examining sources of alternative medicine that are more natural and environmentally friendly antimicrobials, antibiotics, diabetics, antioxidants and crop protection agents. Medicinal plants have provided a good source of a wide variety of compounds, such as phenolic compounds, nitrogen compounds, vitamins, terpenoids, and other secondary metabolites, which are rich in valuable bioactivities, such as antioxidant, anti-inflammatory, antitumor, antimutagenic, anti-carcinogenic, antibacterial, or antiviral activities.

## 3. Cardiovascular Diseases 

CVD was the most common cause of death in 2013, responsible for nearly 31.5% of all global deaths [1]. Approximately 92.1 million U.S. adults suffer from a particular type of CVD. Major causes of CVD are inflammation, increased blood pressure, oxidative stress and many more. This section will introduce three common types of CVD: hypertension, atherosclerosis, and myocardial ischemic reperfusion injury, followed by an introduction of how and which plant- and herb-derived medicines are used to ameliorate the three common types of CVD in the next section [2,3,4]. 

### 3.1. Atherosclerosis 

Atherosclerosis is a disease, in which deposits from cells, inflammation, scar tissues and fat build up in the inner lining of arteries, forming an elementary problem for many CVDs [1]. In the past, it was widely accepted that atherosclerosis was merely an arterial obstruction posed by fatty deposits; recently, researchers have found that atherosclerosis is caused by inflammation, plaque formation as well as modifying vasomotor activity [2,15,16]. 

When exposed to risk factors, for instance, smoking, hypertension or diabetes, endothelial cells lose their function and send signals illustrating injury [2]. In the early stage of life (first decade), “fatty streak” lesions are seen in the aorta [17]. The “fatty streak” lesion is composed of lipids, and this plaque starts an inflammatory response, in which inflammatory cells, such as monocytes and T cells, attach and spread in the endothelium and ultimately to the subendothelial region [2]. Platelets also play a key role in the inflammatory response. Platelets release vasoactive cytokines and chemokines. Inflammatory cells are then released. Stimulated platelets adhere to leukocytes and produce a platelet-leukocyte aggregate that could build up in arteries. These series of endothelial injuries also bring about atypical vasomotor activity. A healthy endothelium generates nitric oxide (NO) by the enzyme NO synthase. NO serves as a vasodilator by controlling smooth muscles as well as platelet aggregation. With the inflammation, reactive oxygen species (ROS) are generated and reduce availability of NO, and conclusively develop atypical vasomotor activity. 

### 3.2. Hypertension 

Hypertension (HTN) is characterized by a systolic blood pressure (SBP) elevated more than 140 mmHg or diastolic blood pressure (DBP) elevated to more than 90 mmHg [1,9]. Two major components that sustain standard blood pressure are cardiac output and peripheral vascular resistance (PVR) [3]. In examining the cause of HTN, it is important to take renal sympathetic nervous system (SNS) into account; renal SNS manages the blood pressure in efferent and afferent levels. The efferent pathway bears the signals from SNS to the kidney, which elevates the renin release, causing the renin-angiotensin-aldosterone system (RAAS) to be activated and heightens the sodium and water retention. Consequently, the volume of circulating fluids is accumulated, which increases the blood pressure. Moreover, the efferent pathway decreases blood flow to the kidney; to compensate for the reduced perfusion, the kidney prompts the afferent pathway and causes overactivity in SNS, continuing the high blood pressure status. 

### 3.3. Ischemia Reperfusion Injury 

Ischemia reperfusion injury (IRI) occurs when tissue is deficient in oxygen, accompanied by reperfusion, triggering an extensive cascade of inflammatory responses that deteriorates surrounding injuries and further into other organs [4]. IRI can be observed during stroke, myocardial infarction (MI) and other parts of the body. Hypoperfusion, or reduced blood flow, often brings unalterable injury in a short period of time, especially for sensitive tissues like myocardium. Blood flow to the oxygen-deficient tissues, if not done in the right time frame, exacerbates the situation by bringing IRI. When examining ischemic tissues without reperfusion, cell death rates were 17% while ischemic tissues that have been exposed to reperfusion had cell death rates of 73% [18]. 

With ischemia, metabolic changes occur because synthesis and degradation of adenosine triphosphate (ATP) is hindered [4]. When tissue is undergoing ischemia, xanthine dehydrogenase is subjected to a structural change to xanthine oxidase that could produce ROS, which could potentially send a cascade down to signal apoptosis. Ischemia also deteriorates antioxidant systems such as dismutase, catalase and glutathione peroxidase, and ultimately heightens the risk for increased amounts of H_2_O_2_ and consequently hydroxyl radicals, which could harm macromolecules in our body, such as lipids, DNA and proteins [18,19]. In response to ischemia, inflammatory cytokines are released, such as TNF-α (Tumor Necrosis Factor-α), IL (Interleukin) and MCP-1 (Monocyte Chemoattractant Protein 1), which are correlated with elevated mortality rates. 

## 4. Introduction of Plant and Herb Derived Medicine

A number of therapeutic medicines derived from plants and herbs exhibit medicinal properties, notably in anti-inflammatory, antioxidant, and anti-hypertensive roles [9,11,20]. This section will provide an overview of how the anti-inflammatory, antioxidant and anti-hypertensive roles in these plants and drugs have made a significant role in treating CVD.

### 4.1. Anti-Inflammatory and Antioxidant Roles of Plant and Herb Derived Medicine

Inflammation is a composite of biological responses that includes microvessels, immune cells, and molecular intermediaries in response to harmful stimuli, such as viruses and damaged cells [21]. Although inflammatory responses can help eliminate damaged cells and repair injured tissues, having pro-inflammatory cytokines and excess ROS released in stress-inducing conditions can lead to inflammatory responses and the progression of diseases [22]. One of the CVD conditions caused by inflammatory responses is atherosclerosis [1]. In order to alleviate the harmful inflammatory responses in the cardiovascular system, such as atherosclerosis, researchers have found various drugs and chemicals containing anti-inflammatory properties; these drugs can inhibit pro-inflammatory cytokines, adhere and penetrate macrophages, and regulate the immunocyte activity [13]. 

Plants are comprised of enzymatic and non-enzymatic antioxidant defense systems that can assist in avoiding the deleterious effects of free radicals [7]. Plants also produce a wide range of molecular weight secondary metabolites, which are imperative in ROS metabolism. ROS takes part in a crucial part of the body in cellular signaling, growth, proliferation and many different activities, but excessive amounts can inflict cell damages that affect the cardiovascular system [23,24]. Further injuries in other organs lead to IRI [4]. However, the onset of injuries can be reduced by phenolic antioxidants, which seem to be the most essential among all secondary metabolites, since they have displayed excellent antioxidant activities in both in vivo and in vitro experiments. Another crucial secondary metabolite is polyphenol, which plays an important role in human health; a high intake has been associated with diminished risks of many chronic diseases, such as cancer, CVD and chronic inflammation [25].

1. Vanillyl alcohol (VA), one of the main phenolic components in plants, is also an active ingredient in *Gastrodia elata Blume*, which had been used as a traditional medicine for centuries [26]. Using fertilized brown Leghorn eggs, Jung and colleagues (2008) discovered that VA can be synthesized from the reduction of vanillin, a commonly used aromatic compound. A study has shown that vanillin, also an active component in *G. elata*, has anti-inflammatory, anti-angiogenic, and anti-nociceptive properties. VA also significantly inhibits the chick chorioallantoic membrane (CAM) angiogenesis, which involves growing new blood vessels from pre-existing vessels [27]. Angiogenesis can cause various conditions, such as metastasis, CVDs, and inflammatory diseases. Down-regulation of angiogenesis could prevent neoplastic growth and inflammation.
2. Curcumin, which is derived from turmeric, consists of antioxidative, anti-apoptotic, and anti-inflammatory properties. It also protects against MI and endothelial injury [28]. Using adult male mouse cardiomyocytes, Wang and colleagues (2017) discovered a novel monocarbonyl curcumin analog, Y20, which has immense curative ability in cardiac injury caused by obesity through anti-inflammatory pathways, portrayed by decreased TNF-α, IL-6, IL-1, and COX-2 (cyclooxygenase-2) levels. Reduction of pro-inflammatory cytokines and COX-2 levels shows that Y20 has a promising therapeutic potential for cardioprotection in obese patients. Moreover, it was found that curcumin downregulates the levels of inflammatory cytokines through inhibition of Egr-1(early growth response-1) in the ischemic heart, which corresponds to reduced TNF-α and IL-6 levels and reduced ischemic injury. Results from Wang and colleagues (2017) suggest that curcumin illustrates anti-inflammatory properties beneficial for the heart via inhibition of pro-inflammatory cytokines, phagocytic cell adhesion and infiltration, and immunocyte activity.
3. Yi-Qi-Fu-Mai (YQFM) is a Chinese herbal medicine, composed of ginseng *Radix Et Rhizoma Rubra* (the root of *Panax ginseng C.A.Mey*.), *Ophiopogonis Radix* (the root of *Ophiopogon japonicas (L.f) Ker-Gawl*), and *Schisandrae Chinensis Fructus* (the fructus of *Schisandra chinensis* (Turcz.) Baill) [29,30]. YQFM successfully suppressed the inflammatory expressions and significantly enhanced myocardial contractility, blood-vessel expansion, anti-lipid peroxidation, and anti-inflammatory effects of the male rats [30]. Another study showed that certain bioactive compounds from Ginseng *Radix Et Rhizoma Rubra*, specifically ginsenoside Rb1, Rg1, and Rg3, can inactivate via NF-kB (nuclear factor kappa-light-chain-enhancer of activated B cells) inactivation and suppress the cytokines. [31]. Ginsenosides also contributes to anti-inflammatory activities. Among the many types of ginsenoside compounds, ginsenoside Ro was classified as the new NF-kB inhibitor [32]. Treating the rats with chronic heart failure (CHF) using YQFM helped to preserve myocardial function, reduce myocardial necrosis, improve cardiac microstructure, decrease brain natriuretic peptide (BNP) levels and relieve inflammation stress. These findings suggest that YQFM exhibits cardioprotective effects in rats with CHF.
4. Xanthone is derived from *G. acuta*, which is a herb that belongs to the Gentianella genus of Gentianaceae family [28]. *G. acuta* can be found in Ewenki medicinal plants in northeast China. Dosage of 400 mg/kg improved heart functions, diminished the oxidative stress of myocardial cells, increased the release of antioxidant enzymes, and prevented mitochondrial dysfunction and myocardial apoptosis against acute IRI. Xanthones also mitigated the release of lactate dehydrogenase (LDH). The levels of LDH and creatine kinase (CK) in the xanthone group were significantly reduced compared with the IRI group (*p* < 0.05). LDH and CK are endoenzymes that are released into the bloodstream during the reperfusion once myocardial cells are degenerated. Therefore, the severity of myocardial damage can be indirectly measured by observing the levels of LDH and CK.
5. Luteolin is mainly derived from flavonoid that not only inhibits tumor development and inflammation, but also suppresses CVD [33]. Luteolin possesses antioxidative, anti-tumor, and anti-inflammatory properties. It also mitigates oxidative stress of the ischemia induced rats through heme oxygenase-1 (HO-1), an integral membrane protein in the smooth endoplasmic reticulum. It is regulated via transcription factors, particularly NF-E2-related factor 2 (Nrf2), which regulates a pathway that promotes cytoprotective effects against oxidative stress. Another way that luteolin blocks oxidative stress is via attenuation of TNF-α levels through NOX4, an isoform of NADPH oxidase for NOX4 expression leads to ROS generation [34]. Luo and colleagues (2017) concluded that luteolin prevents coronary artery disease (CAD), heart failure, and atherosclerosis. Although there has not been a cardiovascular clinical research using luteolin, Luo and colleagues (2017) concluded from an open-label pilot study that 10 mg/kg may become the standard dosage of safety in future CVD studies.
6. Many plants with antioxidant properties contain flavonoid. To evaluate its importance, Jiang and colleagues (2004) extracted flavonoids from *Dracocephalum moldavica* L. (DML) [35]. DML is derived from herbs native to Central Asia. In Xinjiang, China, it is renowned for its medicinal properties in treating stomach and liver diseases, headaches, and congestion. Jiang and colleagues (2004) discovered that the total flavonoids pretreatment significantly reduced the LDH and CK release in rat hearts. To determine the mechanism of flavonoid on cardioprotection, the malondialdehyde (MDA) level, superoxide dismutase (SOD) activity and the ratio of glutathione/glutathione disulfide (GSH/GSSG) were examined in myocardial tissues. In the total flavonoids pretreatment groups, the MDA level decreased considerably while SOD activity and the ratio of GSH/GSSG heightened relative to the group with IRI. Therefore, it was concluded that the total flavonoids attenuate ischemic reperfusion induced enzyme release and one of the mechanisms of the cardioprotection of DML includes antioxidant effects.
7. Grape seed procyanidin extract (GSPE) is a naturally derived compound that possess anti-inflammatory, anti-carcinogenic, and antioxidant characteristics [36]. Data show that GSPE prevents the changes of histopathology and biomarkers in heart, kidney, and liver tissues of the mice exposed to TiO_2_ nanoparticles (NPs); grape seed procyanidin extract prevents the majority of tissue and molecular damage resulting from NP treatment. The protective effect of GSPE may be due to its strong antioxidative activities which are related to the activated Nrf2 and GSPE’s down-regulated genes, including NAD(P)H dehydrogenase[quinine] 1 (NQO1), glutamate-cysteine ligase catalytic subunit (GCLC), and HO-1. Nrf2 expression led to an increase in the oxidative stress and inflammation. The expression of Nrf2 also amplified the damage on the kidneys, which is correlated with induced protein expression of NQO1 and HO-1; expressions of GCLC and HO-1 are associated with increased ROS generation [36,37]. The effect of GSPE has proven beneficial to especially hypercholestrolmeic patients as it has become a renowned herbal supplement for treating CVD.
8. 3,3′-diindolylmethane (DIM) is a plant alkaloid synthesized via hydrolysis of indolylmethyl glucosinolate (glucobrassicin) [38]. To investigate DIM’s antioxidant properties, oxidative stress was induced by doxorubicin (DOX), an antibiotic pertained in anthracycline group that causes DNA intercalation between the base pairs of normal DNA. DIM significantly mitigated DOX-induced oxidative stress by reducing levels of free radicals and lipid peroxidation and increasing the level of reduced glutathione and the activity of antioxidant enzymes. The evaluation of cardiac tissues corroborated the chemoprotective effects of DIM. DIM significantly ameliorated DOX-induced damages, including clastogenicity, apoptosis, and myeloid hyperplasia in bone marrow niche.

### 4.2. Anti-Hypertensive Role of Plant and Herb Derived Medicine

Hypertension (HTN), or high blood pressure, affects 33% of adults in the U.S. population, estimating about 78 million U.S. affected individuals [1]. The rate of HTN has been consistently been rising, with research projection illustrating that by 2030, the rates of HTN could increase to affect about 41.1% adults. It is worthwhile to note that HTN is one of the main causes of CVD and stroke. To alleviate the threatening effects of HTN, researchers have discovered that many medicinal plants, originating in Mexico and East Asia, that control the blood pressure of CVD patients contain an angiotensin-converting enzyme, and vasoprotective effects [9].

1. The traditional Mexican medicine incorporates plants, for instance *B. simaruba*, *J. spicigera*, and *S. lepidophylla*, as anti-hypertensive therapies [39]. These medicinal plants have shown beneficial effects on male rats with glucose-induced hypertension. *B. simaruba* is characterized by negative chronotropic effects (decrease heart rate), long-term hypotension induced by one oral administration of the extract and vasodilating properties that protect the endothelium. *B. simaruba* also has proanthocyanidins, which improve the endothelial function through vascular endothelial NO synthase activation, thus explaining the vascular protecting effect. *J. spicigera* includes eucalyptol as one of its main ingredients, which promotes vascular smooth muscle relaxation and creates anti-hypertensive effects. *S. lepidophylla* promotes diuresis and includes biflavonoids, which could also produce anti-hypertensive effects.
2. Qishenyiqi (QSYQ) is a Chinese traditional medicine that was often used to treat CHD and CHF [40]. It is composed of six Chinese herbs: Radix Astragali Mongolica, *Salvia miltiorrhiza* bunge, Flos Lonicerae, Poria, Radix Aconiti Lateralis Preparata, and Radix Glycyrrhizae. Qiu and colleagues (2014) used cardiac tissues from miniature pigs and discovered that QSYQ had improved heart function in pigs with CHF. QSYQ can ameliorate and remodel any abnormal enlargement in myocardium by inhibiting the Angiotensin II (Ang II) expression in LAD (left anterior descending coronary) of rats. Ang II is one of the main peptides in the renin-angiotensin system. Ang II biological effects include blood vessel contraction, fibrinolysis inhibition, and tissue fibrosis promotion. QSYQ can diminish these effects by reducing apoptosis and decreasing the level of TNF-α and active caspase-3. Caspase-3 plays an important role as a substrate of apoptosis and has been used as a sign of irreversible apoptosis. In addition to apoptosis-induced biological effects, caspase-3 can also shear the muscle fibers cells α-actin and troponin T, and α-actin and muscle fiber rupture further reduce contractile function.
3. Guanxin Danshen Formulation (GXDSF) is a Chinese herbal medicine that is comprised of *Salviae miltiorrhizae Radix et Rhizoma*, *Notoginseng Radix et Rhizoma*, and *Dalbergiae odoriferae Lignum* [41]. Deng and colleagues (2017) confirmed the effects of GXDSF on myocardial ischemia reperfusion injury-induced left ventricular remodeling (MIRI-LVR) rats, indicating that GXDSF has a cardioprotective effect on CVD. They also discovered that the GXDSF treated group had lower levels and increased ratio of collagen I and (*p* < 0.05), suggesting that GXDSF exhibits some anti-fibrosis effects. Collagen subtypes I and III are the main constituents in interstitial collagens that influence cardiac function. More importantly, measuring the collagen III/I ratio helps to determine the effectiveness of GXDSF [42]. In addition, the GXDSF-treated group had lower fibrosis conditions, suggesting that GXDSF can inhibit ventricular fibrosis in the mouse heart.
4. Ethanolic extract from the root bark of Ulmus macrocarpa (RBUM) has been used as an Chinese traditional medicine for treating inflammation, edema, mastitis, gastric cancer [43]. RBUM’s active ingredients include procyanidin oligomers, which are a subclass of flavonoids that include catechin oligomers, a natural phenolic antioxidant that protects against cadmium nephrotoxicity [44]. The procyanidin oligomers have shown antioxidant, anti-inflammatory, and anti-hypertensive properties; procyanidin oligomers are present in several plants, such as grapeseed. Oh and colleagues (2008) discovered that oral administration of 100 mg/kg RBUM decreased the systolic blood pressure in spontaneously hypertensive male rats by approximately 20 mmHg, compared to the vehicle treated group. The anti-hypertensive effect of RBUM may be due to its ability to recover structural and functional changes of the vascular endothelium in SHR (spontaneously hypertensive rats) [43].

## 5. Synergistic Effects of Nanoparticles Combined with Plant and Herb Derived Medicine

As aforementioned, plant- and herb-derived medicines are widely used for their low toxicity and compatibility with the human body [8]. However, plant- and herb-derived medicines often need to be combined with other synthetic particles, or NP, to supplement for its setbacks, such as poor solubility [45]. Although NP usage in theragnostic purposes for cancer has been explored, cardiovascular applications have been less researched [46]. Cardiovascular applications of NP have illustrated great promise for its targeted drug delivery system. This section will introduce NP and its combination with plant and herb derived medicine for CVDs.

1. Total flavonoid extract from *Dracocephalum moldavica* L. (TFDM) is composed of *Dracocephalum moldavica* L., a plant in the Labiatae family. TFDM is known to have cardioprotective qualities against CHD, HTN and atherosclerosis [45]. The specific agent that is accountable for its pharmacological effect comes from the flavonoid extract from the plant. Despite its beneficial pharmacological properties, like many flavonoids, TFDM has low solubility and bioavailability. In order to maximize its cardioprotective benefits, solid lipid nanoparticles (SLNs) were incorporated. SLNs are taken into intestinal epithelial cells of male rats after lipolysis and improve the bioavailability and solubility of TFDM. It was found that TFDM-SLN, compared to just TFDM, had a 5.72% increase in its drug release as well as significantly decreasing the MI size and improving myocardium integrity. TFDM-SLN also reduced the amount of IL-1β and TNF-α, illustrating an anti-inflammatory trait.
2. Selenium-incorporated guar gum (GG) NPs have also shown cardioprotective activities regarding IRI [47]. Selenium is a powerful antioxidant that has low toxicity. Furthermore, selenium NPs have shown exceptional bioactivity and bioavailability and even less toxicity than sodium selenite, the dietary form of selenium. GG is an easily attainable polysaccharide from the *Cyamopsis tetragonoloba* seed. GG is a good source of fiber and assists in lowering cholesterol levels. To enhance its cardioprotective properties, selenium incorporated guar gum (SGG) NPs were made to examine its antioxidant properties. When compared to guar gum nanoparticle (GGN) and selenium NP used independently, SGG NP illustrated improved antioxidant properties such as enhanced reducing power, metal chelation abilities as well as hydroxyl radical scavenging effects in rat cardiomyoblasts (H9c2). Furthermore, SGG portrayed considerable protective traits in IRI in H9c2 cells through its antioxidant characteristics.
3. PVAX, or copolyoxalate containing VA, is made up of H_2_O_2_-targeting peroxalate ester linkages covalently bonded to the backbone, incorporated with VA [48,49,50]. VA, as previously mentioned, is from *Gastrodia elata* Blume, an herbal factor commonly used for ischemic injury and CVDs; VA is known for its antioxidant and anti-inflammatory activities. VA and peroxalate ester bond together; together they are excellent H_2_O_2_ scavengers that prevent ROS production synergistically in male rats. PVAX’s anti-inflammatory characteristics were conveyed by the reduction of pro-inflammtory cytokines and MI size. It also illustrated reduction of NOX2 and NOX4, which consequently assist in lowering ROS generation after IRI.

## 6. Conclusions 

From prehistory up to the modern day, herbal and plant-based medicines have taken a great part in attending to our cardiovascular health [10]. CVDs such as atherosclerosis, HTN or IRI are expected to continually rise at unprecedented rates in the coming years [1]. With the elevating rates of CVD, exploration of herb- and plant-derived medicine with antioxidant, anti-inflammatory and anti-hypertensive properties as well as efficacy of these medicines in humans is crucial to further assess the biocompatibility of naturally derived medicine in humans [7,8,9]. Furthermore, it is important to delve deeper into natural derivatives and their potential with nanoparticles for remedies for synergistic effects of modern improvement of technology and naturally derived medicine [46]. 
